# Multiple dyes applications for fluorescent convertible polymer capsules as macrophages tracking labels

**DOI:** 10.1016/j.heliyon.2024.e30680

**Published:** 2024-05-05

**Authors:** Zhanna V. Kozyreva, Polina A. Demina, Anastasiia Yu Sapach, Daria A. Terentyeva, Olga I. Gusliakova, Anna M. Abramova, Irina Yu Goryacheva, Daria B. Trushina, Gleb B. Sukhorukov, Olga A. Sindeeva

**Affiliations:** a Vladimir Zelman Center for Neurobiology and Brain Rehabilitation, Skolkovo Institute of Science and Technology, 30 b.1 Bolshoy Boulevard, 121205, Moscow, Russia; b Science Medical Center, Saratov State University, 83 Astrakhanskaya Str., 410012, Saratov, Russia; c Institute of Molecular Theranostics, Sechenov University, 8-2 Trubetskaya Str., 119991, Moscow, Russia; d School of Engineering and Materials Science, Queen Mary University of London, Mile End Road, London, E1 4NS, UK; e Life Improvement by Future Technologies (LIFT) Center, Skolkovo, 143025, Moscow, Russia

**Keywords:** Microcapsule, Encapsulation, Fluorescent label, Photoconvertible label, Photoconversion, Carbon nanoparticle, Deethylation, Cell imaging, Cell labeling, Macrophage

## Abstract

Tracing individual cell pathways among the whole population is crucial for understanding their behavior, cell communication, migration dynamics, and fate. Optical labeling is one approach for tracing individual cells, but it typically requires genetic modification to induce the generation of photoconvertible proteins. Nevertheless, this approach has limitations and is not applicable to certain cell types. For instance, genetic modification often leads to the death of macrophages. This study aims to develop an alternative method for labeling macrophages by utilizing photoconvertible micron-sized capsules capable of easy internalization and prolonged retention within cells. Thermal treatment in a polyvinyl alcohol gel medium is employed for the scalable synthesis of capsules with a wide range of fluorescent dyes, including rhodamine 6G, pyronin B, fluorescein, acridine yellow, acridine orange, thiazine red, and previously reported rhodamine B. The fluorescence brightness, photostability, and photoconversion ability of the capsules are evaluated using confocal laser scanning microscopy. Viability, uptake, mobility, and photoconversion studies are conducted on RAW 264.7 and bone marrow-derived macrophages, serving as model cell lines. The production yield of the capsules is increased due to the use of polyvinyl alcohol gel, eliminating the need for conventional filtration steps. Capsules entrapping rhodamine B and rhodamine 6G meet all requirements for intracellular use in individual cell tracking. Mass spectrometry analysis reveals a sequence of deethylation steps that result in blue shifts in the dye spectra upon irradiation. Cellular studies on macrophages demonstrate robust uptake of the capsules. The capsules exhibit minimal cytotoxicity and have a negligible impact on cell motility. The successful photoconversion of RhB-containing capsules within cells highlights their potential as alternatives to photoconvertible proteins for individual cell labeling, with promising applications in personalized medicine.

## Graphical abstract

**Figure:**
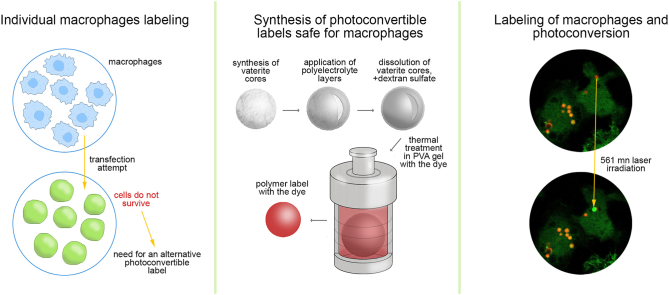

## Nomenclature

Rhodamine B: (RhB)
rhodamine 6G: (Rh6G)
pyronin B: (PyB)
fluorescein: (Fl)
acridine yellow: (AY)
acridine orange: (AO)
thiazine red: (TR)
polyvinyl alcohol: (PVA)
green fluorescent protein: (GFP)
bone marrow-derived macrophages: (BMDM)
scanning electron microscopy: (SEM)
confocal laser scanning microscopy: (CLSM)
ultraviolet: (UV)

## 1 Introduction

Cell tracking plays a crucial role in understanding the behaviour, migration, and fate of individual cells within large populations [[1], [2], [3], [4]]. The possibility to trace cell pathways provides valuable insights into various biological processes, such as the interactions between cancer cells and immune cells [5]. This allows for the evaluation of the proliferation degree and rate for further selection of immune cells with the best characteristics. The subsequently created cell line derived from cell selection and purification holds potential for applications in tissue engineering and personalized medicine, specifically for targeted drug delivery to the tumor site.

Optical labeling of cells has emerged as a powerful tool for cell tracking, offering the potential to label and monitor cells with high precision. This approach primarily relies on the use of genetically modified cells expressing green fluorescent proteins (GFP-like proteins) such as Dreiklang [6], Dronpa [7], mTFP0.7 [8], rsCherryRev [9], IrisFPs [10], and others. The synthesis of GFP-like proteins in cells is achieved via transfection [11]. Such genetic modification is effective for certain cell types, for example, fibroblasts [12], chinese hamster ovary (CHO) cells [13], astrocytes [14], neural cells [15], and others. Although, other cell types such as primary cultures [16], especially stem cells [17] and macrophages [18] are problematic for gene modification and hence their tracking is challenging. Particularly for macrophages, transfection often results in cell death due to the immune response triggered by foreign nucleic acids [19]. Consequently, the development of macrophage cell lines with genetically encoded fluorescent proteins for labeling purposes has become a challenging task [18]. Furthermore, the process of transfection gives rise to concerns regarding the potential for utilizing these cells in the scope of personalized medicine. The discussed challenges limit the applicability of traditional optical labeling techniques with GFP-like proteins.

To search for an alternative to transfection, the utilization of polymer microcapsules was proposed in previous studies [20]. Capsules function as microreactors that combine fluorescent dye rhodamine B (RhB) and carbon nanodots (CNDs) as a photocatalyst. Upon subjecting the polymer capsules to laser radiation, a photocatalytic reaction occurs within the capsule, resulting in alterations in the emission spectra of RhB. Polymer capsules are highly advantageous as tracking labels because of their low toxicity [21], ease of internalization by diverse cell types, and prolonged retention within cells [22]. These properties make them a desirable option for cell tracking purposes. Nevertheless, there are still some challenges to this approach. One of these is a need to explore additional fluorescent dyes that emit in different spectral ranges, in order to expand the possibilities for cell tracking. The use of distinct colors for convertible capsules is crucial for tracking multiple cell populations within the same region and for investigating their behavior and interactions. Furthermore, it is essential to select dyes with non-overlapping spectral ranges for capsules and other dye-based cell-staining techniques. Another unresolved issue pertains to the significant loss of capsules during hydrothermal treatment. It happens primarily due to capsules melting together when synthesis is performed in a dye-aqueous solution. Subsequent filtration to remove fused capsules further exacerbates capsule loss.

This study aims to develop a safe, efficient, stable, scalable, and easy-to-reproduce technique for labeling and tracking macrophages as an alternative to GFP-like proteins. The possibility of scaling up the yield from one synthesis was verified using polyvinyl alcohol (PVA) gel as a reaction medium. Rhodamine 6G (Rh6G), pyronin B (PyB), fluorescein (Fl), acridine yellow (AY), acridine orange (AO), and thiazine red (TR) were investigated in comparison to previously reported RhB [20]. Capsules with various dyes were examined for their brightness of fluorescence, photostability, and photoconversion ability with confocal laser scanning microscopy (CLSM). The photoconversion mechanism was studied using mass spectrometry. RAW 264.7 and bone marrow-derived macrophages (BMDM) served as model macrophage lines to evaluate the safety and labeling efficacy of polymer capsules. Viability assay, uptake measurements, and scratch assay were performed. The final task of this research was focused on the photoconversion of microcapsules inside macrophages and the assessment of cell viability after the procedure.

## 2 Materials and methods

### 2.1 Materials

Poly(allylamine hydrochloride) (PAH, Mw = 17.5 kDa), poly(4-styrene sulfonate) sodium salt (PSS, Mw = 70 kDa), calcium chloride dihydrate, sodium carbonate, sodium chloride, ethylenediaminetetraacetic acid (EDTA), dextran sulfate sodium salt (DS, Mw = 40 kDa), rhodamine B, rhodamine 6G, pyronin B, fluorescein, acridine yellow, acridine orange, thiazine red, and polyvinyl alcohol were purchased from Sigma.

Dulbecco's modified Eagle's medium (DMEM), Dulbecco's modified Eagle's medium F12 (DMEM F12), phosphate-buffered saline (PBS), fetal bovine serum (FBS), trypsin EDTA, bovine serum albumin (BSA), calcein-AM ﬂuorescent dye, hoechst 33258 dye, dimethyl sulfoxide (DMSO) and MTT cell proliferation assay ((3-[4,5-dimethylthiazol-2-yl]-2,5 diphenyl tetrazolium bromide) assay) kit were purchased from Thermo Fisher Scientific.

Deionized (DI) water (resistivity 18.2 MΩ cm at 25 °C) from the Direct-Q water puriﬁcation system was used to prepare all solutions.

### 2.2 Fabrication of fluorescent polymer capsules by thermal treatment

Capsules were prepared analogically to the procedure described by Demina et al. [20] with some modifications. To synthesize layer-by-layer polymer capsules, calcium carbonate microparticles (vaterite) were prepared. Equal volumes (0.650 μL) of 1 M calcium chloride and 1 M sodium carbonate were combined under constant stirring in 3 mL of water. The obtained particles were washed twice with water and once with ethanol, and were left to dry in a binder drying oven at 60 °C for 3 h. Then, polyelectrolytes were applied alternating PAH and PSS (1 mg/mL in 0.15 M NaCl). Each layer was adsorbed onto the vaterite particles for 10 min at the rotator and washed three times with water using centrifugation (1400×*g*, 2 min). Consequently, four PAH/PSS bilayers were obtained. EDTA 0.2 M solution was used to dissolve the CaCO_3_ cores. Subsequently, a DS aqueous solution (2 mg/mL) was added to the capsules, and the sample was left on the rotator for 1 h. The suspension was washed with water three times using centrifugation and dispersed in a dye-aqueous solution. Concentrations were different for each dye depending on their solubility: RhB, Rh6G, AO, and TR – 0.5 mg/mL; AY – 0.06 mg/mL; Fl and PyB – 0.05 mg/mL. Then, the capsules were mixed in PVA water gel (the final concentration of PVA in the gel was 15 %) and placed in a high-pressure autoclave for thermal treatment (180 °C, 3 h). After cooling, the capsules were washed in DI water several times until the PVA gel was removed (5600×*g*, 4 min).

### 2.3 Scanning electron microscopy (SEM)

SEM images were obtained using a VEGA 3 LM, TESCAN (Czech Republic, Brno). SEM was utilized to assess the morphology of capsules before and after thermal treatment and to calculate the diameter of the capsules. The samples were prepared by depositing a drop of the capsule suspension on copper tape and leaving it to dry at room temperature. Before imaging, the samples were coated with a thin gold ﬁlm (approximately 5 nm thick) using a Q150R ES Plus rotary-pumped coater (Quorum, England). SEM images analysis was performed using the ImageJ software.

### 2.4 Confocal laser scanning microscopy (CLSM)

CLSM was performed using a confocal laser scanning microscope Leica TCS SP8 X (Leica, Germany) equipped with an objective 20×\0.70 NA. CLSM was used to visualize capsules, detect changes in capsules' fluorescence intensities, and assess the photoconversion ability of labels. Lasers with wavelengths of 405, 488, and 561 nm were used for microcapsule excitation and detection. The laser power densities for the visualization of the samples were 6 kW/cm^2^ for RhB, 18 kW/cm^2^ for Rh6G and PyB, and 46 mW/cm^2^ for Fl, AY, AO, and TR. The laser power density used for the photoconversion process was 451 kW/cm^2^.

The ﬂuorescence of the RhB, Rh6G, and PyB dyes was excited at 561 nm and detected in the range of 580–620 nm for RhB and the range 580–670 for Rh6G and PyB (red channel). The fluorescence of the capsules with dyes Fl, AY, AO, and TR was excited at 488 nm and detected in the range 505–600 nm (green channel). The photostability of the capsules containing the dyes was evaluated by collecting a time series of 15 scans at standard settings used for cell imaging. The settings included a 1024 × 1024 pixel spatial resolution, a 100 Hz scanning speed, and adjusted laser intensities. The λ-scans were recorded for capsules by excitation with 488 and 405 nm lasers. The selected scan ranges were 505–700 and 420−600 nm, respectively, with a 20 nm detection bandwidth and a 5 nm λ-detection step size. For the photoconversion procedure, the smallest possible scanning area was chosen, which corresponded to 12.11 × 12.11 μm with 512 × 512 pixels and a scanning speed of 10 Hz. Changes in the fluorescence intensities of the capsules were evaluated using Gwyddion software.

### 2.5 Mass spectrometry

Mass spectrometry was performed using a Q Exactive Hybrid Quadrupole-Orbitrap (Thermo Fisher Scientific, USA). Mass spectrometry was used to evaluate the effect of ultraviolet (UV) radiation on the chemical structure of the dyes in the solution after thermal treatment as an analog to the photoconversion procedure in capsules. RhB and R6G were prepared in two forms. The first was an initial solution of the dye in water (0.24 mM). The second form was a solution of the dye at a concentration of 0.6 mM, autoclaved with DS (0.2 μg/mL) for 3 h at 180 °C. DS was added at such concentration that there were 1000 molecules of the dye for each obtained carbon dot. It was calculated using information on the average size of the CNDs [23]. These concentration requirements were met to avoid off-scale from CNDs. The prepared solutions were diluted 2.5 times with water, poured into quartz cells, and placed in the dark chamber. In the chamber, the solution was irradiated with a UV lamp (15 W, 254 nm peak) for 3 h under constant stirring. Before the mass spectrometry measurements, the samples were diluted twice with a solution of equal parts of acetonitrile and water with 0.1 % formic acid. The injection volume was 200 μL. Ionization was performed with electrospray in positive mode. The detection range was 200–1000 *m*/*z*.

### 2.6 Cell experiments

#### 2.6.1 Cell preparation

The mouse monocyte/macrophage-like cells RAW 264.7 were purchased from ATCC (No. TIB-71). RAW 264.7 cell line was cultivated at 37 °C under 5 % CO_2_ in DMEM culture medium containing 10 % FBS and 1 % antibiotics (100 U/mL penicillin/100 μg/mL streptomycin sulfate). The culture medium was replaced every 3–4 days and the cells were detached using a plastic cell scraper. Bone marrow cells were used to generate BMDM as previously described [24] using an L929-cell conditioned medium as a source of macrophage colony-stimulating factor [25]. The mice were euthanized by cervical dislocation, and the femurs and tibia were removed, washed in sterile PBS with antibiotics, and severed proximal to each joint. The bone marrow was rinsed with PBS using a syringe with a 25G needle, centrifuged for 10 min at 500 g, resuspended in DMEM-F12 containing 20 % L929 culture medium, and plated on a plastic flask. Bone marrow cells were incubated at 37 °C in 5 % CO_2_ for at least seven days, after which the macrophages were harvested and used for experiments.

#### 2.6.2 MTT assay

The cell culture was plated on a 96-well plate at a density of 1 × 10^4^ cells/well for RAW 264.7 and 5 × 10^4^ cells/well for BMDM and incubated overnight in a humid atmosphere of 5 % CO_2_ at 37 °C. Then, four samples of polymer capsules (1, 5, and 10 capsules/cell) were added to the wells and incubated for 48 h. The samples included empty, heat-treated empty, and heat-treated capsules with RhB or Rh6G dyes. Then, the culture medium was replaced with 100 μL of freshly prepared 0.05 % (w/v) MTT solution in the medium and incubated for 3 h at 37 °C. The medium was then replaced with 100 μL of DMSO, and the plate was left for 15 min on a shaker to dissolve formazan crystals. As a control, cells without the addition of polymer capsules were used. The absorbance of each well was measured at 570 nm wavelength using a single-mode microplate reader (Infinite® M Nano, TESCAN, Czech Republic, Brno).

#### 2.6.3 Uptake measurements

The cell cultures were plated on a 24-well plate at a density of 5 × 10^4^ cells/well and incubated overnight in a humid atmosphere of 5 % CO_2_ at 37 °C. After incubation, thermally treated polymer capsules with RhB were added to the wells (1, 5, and 10 capsules/cell) and incubated for 24 h. Then, the culture medium was removed and cells were washed three times with PBS to remove non-internalized capsules. 500 μL of fresh medium was added. For cell staining, 1 μL of 10,000 times diluted calcein-AM and hoechst 33258 (1:10000) stock solutions were added to each well and incubated for 30 min. After incubation, the growth medium was removed and the cells were washed with PBS and harvested using a small spatula. Then, the cells were transferred into PBS with 2 % FBS solution and measured using an ImageStream X Mark II Imaging flow cytometer (Luminex, USA). The measurement was performed using the INSPIRE software with the following equipment settings: 40× magnification, low flow rate/high sensitivity, 488 nm laser, and 50 mW laser power. Uptake investigations for each cell line were performed in three replicates for each concentration of added capsules. Each replicate involved the analysis of 1000 objects. The number of internalized capsules was determined using the Spot Count feature of the IDEAS software (Luminex, USA).

#### 2.6.4 Dyes encapsulation stability in cells

Cell cultures were plated on a 24-well plate at a density of 35 × 10^4^ cells/well and incubated overnight in a humid atmosphere of 5 % CO_2_ at 37 °C. Then, capsules containing RhB and Rh6G were added at a concentration of 5 capsules/cell. Images of the cells were obtained 0.5, 2, and 24 h later using an inverted luminescent microscope NIB-FL (LOMO-Microanalysis, Russia). For the fluorescence spectra measurements, RhB and Rh6G aqueous solutions (0.4 μM) were mixed either with water or with BSA aqueous solution (1.5 μM) and placed in a thermoshaker for 2 h at 37 °C. The fluorescence spectra were recorded using a single-mode microplate reader.

#### 2.6.5 Cell mobility test

RAW 264.7 and BMDM cells were plated on a 24-well plate at a density of 35 × 10^4^ cells/well and incubated overnight in a humid atmosphere of 5 % CO_2_ at 37 °C. Then, capsules with RhB were added at a concentration of 5 capsules/cell and incubated for 2 h. Then, the pipette scratch method was performed, as described previously [26]. After the scratch, the cells were washed three times with PBS to remove detached cells and non-internalized capsules. Images of cell migration were obtained with an inverted luminescent microscope immediately after the scratch, 24 and 48 h later. The migration stage was evaluated by calculating the area between the edges of the wound using ImageJ software.

#### 2.6.6 Photoconversion of capsules inside the cells

Cell lines were plated on a 35 mm Glass bottom dish with a 14 mm micro-well at a density of 15 × 10^4^ cells per dish in a complete culture medium (10 % FBS) and transferred to a CO_2_ incubator overnight. The next day, five capsules per cell were added to the cells, and the cells were incubated with the sample for 24 h. Non-internalized capsules were washed away with PBS. To visualize the cytoplasm, the samples were incubated with calcein-AM (1:3000), followed by washing with PBS. Photoconversion of capsules containing RhB inside the cells was performed at the same setting of laser parameters and detection regions used previously (subsection 2.4).

## 3 Results and discussion

### 3.1 High yield production of fluorescent capsules synthesis via PVA gel

A recent study [20] revealed that carbonized polyelectrolyte capsules containing RhB retained high fluorescence after thermal treatment, exhibited photoconversion properties, and were stable for cell tracking. This type of capsules was prepared according to a previously described method based on PSS and PAH polymers [27]. DS was also applied to the capsules as a source of CNDs, which were formed during thermal treatment and played the role of a catalyst for the photoconversion of the dye during laser irradiation [28]. The dye encapsulation in capsules was performed through hydrothermal synthesis in water, which presented certain limitations. The capsules sank to the bottom of the autoclave and formed a single layer. If too many capsules were placed in the autoclave, capsules would remain in contact, leading to their fusion at high temperatures (Fig. S1a). Therefore, an additional filtration step was required to remove large aggregates. Both the limit of capsule load in the autoclave and the filtration step caused a reduction in the production yield.

**Fig. 1:**
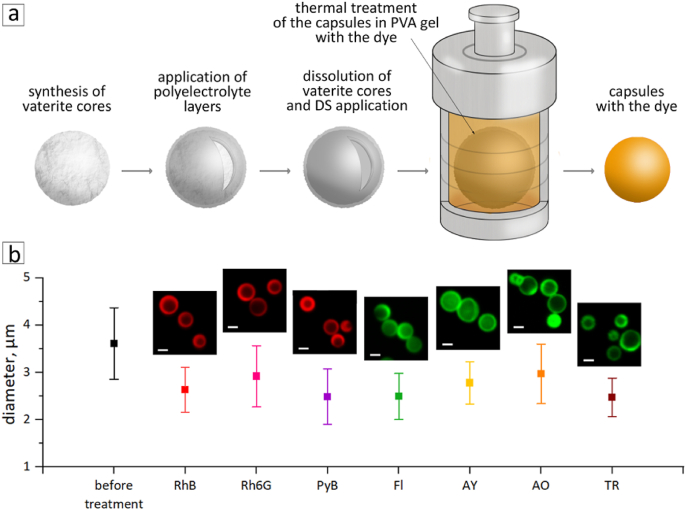
(a) Scheme of the dye-loaded thermally treated capsules preparation in PVA gel. (b) Graph of the capsule diameters before and after thermal treatment with the dyes RhB, Rh6G, PyB, Fl, AY, AO, and TR with corresponding CLSM images (scale bar is 2 μm).

Therefore, in the first stage of this research, the last step of the synthesis was modified to increase the production yield of capsules (Fig. 1a). During the hydrothermal treatment, 15 % PVA gel was used as the medium for capsules instead of water. The capsules were thoroughly mixed with the chosen dye in the PVA gel. The appropriately high viscosity of the gel [29] allowed the capsules to remain separate from each other in the thickness of the gel. This prevented the capsules from fusing during autoclaving and eliminated the need for further filtration (Fig. S1b). In addition, more capsules could be placed in the autoclave in this approach because capsules were distributed in the volume of the reactor and not only at the bottom. After complete cooling, the PVA gel was removed by washing the capsules several times with water and centrifugation. As a result, the capsule yield in one synthesis using PVA gel increased by at least 250 times in comparison to the synthesis in water.

### 3.2 Encapsulation of various fluorescent dyes

In addition to RhB, other dyes have been reported to exhibit blue spectral shifts upon irradiation. These dyes include Rh6G [30], PyB as an analogue of rhodamine dyes [31], Fl [32], AY [33], AO [34], and TR [35]. These dyes were selected for further investigation in this research as promising candidates for the synthesis of photoconvertible labels. The objective was to assess the potential of loading these dyes into capsules while preserving their inherent fluorescent properties.

SEM images of the capsules before and after treatment in the PVA gel for each of the seven dyes were obtained (Fig. S2). The diameters of the capsules were calculated using ImageJ based on at least 150 capsules (Fig. S3). It turned out to be 3.6 ± 0.8 μm for the initial capsules and approximately 75 % less for the capsules after thermal treatment (Fig. 1b). The decrease in capsule size due to the thermal treatment was also observed by other researchers [36]. This is related to the increased mobility of the polymer chains and hydrophobic interactions. In addition to the decrease in size, SEM also demonstrated a change in the capsules’ shape. Thus, dried capsules without hydrothermal treatment collapsed due to the absence of a hardcore. At the same time, capsules after heat treatment retained a spherical shape, which also indirectly indicates compaction of the polymer layers and an increase in the thickness of the shell.

CLSM images of the capsules are presented above each size data point (Fig. 1b). The excitation and emission parameters were tuned based on the possibility of locating them as close as possible to the fluorescence spectra of dyes. For capsules containing RhB, fluorescence was excited by a 561 nm laser and detected in the range of 580–620 nm. Rh6G and PyB were excited by a 561 nm laser and detected in the range of 580–670 nm, which is referred to as the red channel for ease of perception. Fl, AY, AO, and TR were excited by a 488 nm laser and detected in the range 505–600 nm, which is referred to as the green channel. The applied excitation wavelengths and detection ranges of the capsules conformed to those of the dye excitation and emission spectra (Fig. S4), except for TR.

TR has an excitation wavelength maximum of 509 nm and an emission wavelength maximum of 607 nm (Fig. S4). Therefore, it is supposed to be excited with a 561 nm laser and visualized in the red channel. Nevertheless, capsules with TR were detected in the green channel. Most probably, such a change in fluorescence parameters is related to the effects of high temperature and pressure during hydrothermal synthesis, which indirectly indicates a change in the structure of the dye. Since capsules containing TR still exhibited noticeable fluorescence in the green channel, the sample was opted for further investigation. Thus, temperature treatment of microcapsules during hydrothermal synthesis provided the encapsulation of a wide range of low molecular weight dyes due to the ability of polyelectrolyte layers to compress under high temperatures, which is consistent with the results of other authors [37]. Note that encapsulated dyes retained their fluorescent properties while only TR changed its spectral properties after encapsulation (Fig. 1b).

### 3.3 Fluorescence intensity of capsules containing various dyes

**Fig. 2:**
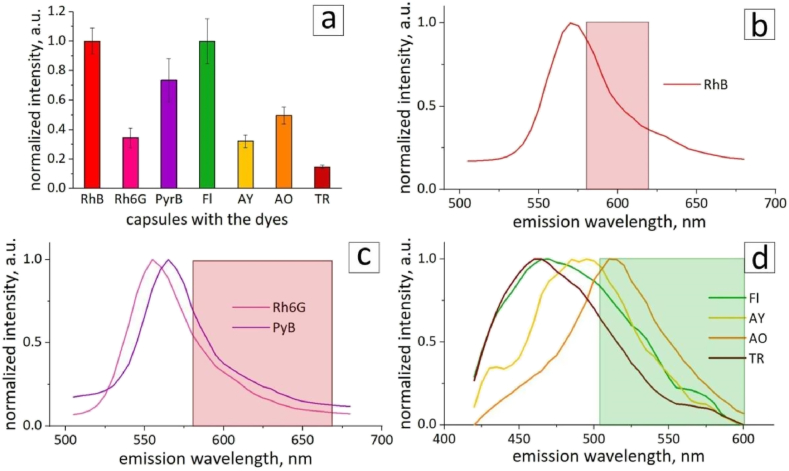
(a) Angularly averaged fluorescence intensities of the capsules with dyes RhB, Rh6G, and PyB (detection range 580–620/670 nm, red channel) and Fl, AY, AO, and TR (detection range 505–600 nm, green channel). λ-scans of capsules with (b) RhB, (c) Rh6G, and PyB (488 nm excitation). λ-scans of capsules with (d) Fl, AY, AO, and TR (405 nm excitation). Red and green rectangles correspond to the detection ranges 580–620/670 nm and 505–600 nm, respectively.

Stable and bright fluorescence of labels is a vital factor for prolonged cell tracking. Therefore, the fluorescence intensity values of the capsules were carefully analyzed. This was performed by obtaining a series of angularly averaged intensity profiles from CLSM images. Images were obtained with equal laser settings in separate channels (except for RhB, which is further explained). The obtained values were normalized separately for the dyes in the red (RhB, Rh6G, and PyB) and green (Fl, AY, AO, and TR) channels (Fig. 2a). The fluorescence intensities of the capsules varied across the samples. The difference in intensity is related to the excitation and detection conditions of the dyes. λ-scans are presented in Fig. 2b–d. Red rectangles correspond to the 580−620/670 nm detection ranges (red channel) and green rectangle corresponds to the 505−600 nm detection range of the confocal microscope (green channel). These parameters were set to obtain confocal images. Such parameters were less optimal for Rh6G, PyB, AY, AO, and TR, as can be seen from their λ-scans, but could not be changed due to the discreteness of the lasers included in the design of the confocal microscope. RhB exhibited the highest intensity among the dyes in the red channel. Therefore, a lower laser power density and a narrower detection region were chosen for this dye (Fig. 2b). Fl exhibited the highest fluorescence intensity in the green channel. Its emission maximum was located farther from the detection region than those of AY and AO (Fig. 2d). Nevertheless, it has a higher quantum yield (0.79 against 0.47 and 0.2 for AY and AO, respectively) [[38], [39], [40]], therefore, its intensity is higher. It is usually sufficient to increase the laser intensity and gain to obtain bright confocal images to overcome the weak fluorescence. Nevertheless, it would have had a high impact on the stability of the dyes, which was further demonstrated.

### 3.4 Study of photostability of dyes in capsules

**Fig. 3:**
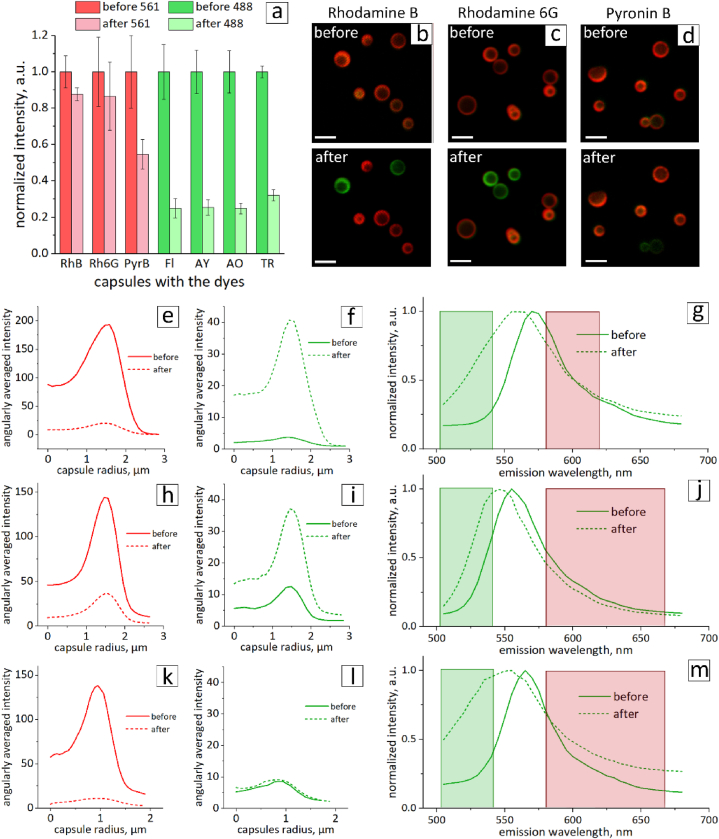
(a) Normalized changes in the fluorescence intensity of the dyes before and after the bleaching test. CLSM images of thermally treated microcapsules in PVA gel with RhB (b), Rh6G (c), and PyB (d) before and after photoconversion (scale bar is 5 μm). Changes in the angularly averaged fluorescence intensity of the capsules before and after laser irradiation in the red and green channels of RhB (e,f), Rh6G (h,i), and PyB (k,l). Capsule emission spectra under 488 nm excitation before and after laser conversion for RhB (g), Rh6G (j), and PyB (m) (green and red rectangles correspond to the 505–540 and 580–620/670 nm detection ranges of the confocal microscope, respectively).

One of the most important characteristics of cell labels is photostability during tracking for several days. It is crucial for the fluorescent label to withstand prolonged detection and imaging processes. To evaluate the photostability of the dyes in the capsules, bleaching tests were performed. Time-series confocal scans of 15 frames were obtained at standard laser settings used to record high-quality images of the cells. The fluorescence intensities of the capsules before and after the time series were calculated by angularly averaging the intensity profiles. The intensities are presented in Fig. 3a and were normalized to the data before the bleaching test.

According to the obtained graph, capsules containing RhB and Rh6G lost approximately 10 % of their fluorescence intensity after the procedure. PyB dye intensity was reduced by 40 %. Fl, AY, AO, and TR lost approximately 75 % of their fluorescence intensity. This means that even if the laser intensity and gain are increased to obtain brighter confocal images, it will lead to even more rapid bleaching of these dyes. Low fluorescence intensity and photostability shown in dyes Fl, AY, AO, and TR made them unfavorable for long-term tracking. Therefore, these four dyes were excluded from further studies. The following experiments were performed on capsules containing RhB, Rh6G, and PyB dyes.

### 3.5 Photoconversion on capsules with RhB, Rh6G, and PyB

The photoconversion abilities of capsules containing RhB, Rh6G, and PyB were analyzed. CLSM images before and after the photoconversion procedure are presented in Fig. 3b–d. The ﬂuorescence of the capsules was excited at 561 nm and detected in the range 580–620/670 nm. Photoconversion was performed using a laser with a wavelength of 561 nm. The increased signal after photoconversion appeared in the range 505–540 nm for RhB and Rh6G excited by the laser with a 488 nm wavelength. At the same time, no such effect was observed for PyB.

For each sample, angularly averaged profiles of the fluorescence intensities were extracted from the CLSM images before and after the procedure. The intensities changed both in the green and red channels for RhB and Rh6G (Fig. 3e,f,h,i). The fluorescence signal significantly reduced in the red channel and considerably increased in the green channel. Thus, the desired photoconversion effect was observed due to changes in the intensity of the signal in red and green channels. The photoconversion of capsules with RhB correlates with the data obtained earlier for capsules with this dye made by hydrothermal treatment in water [20]. Both RhB and Rh6G belong to the rhodamine family [41]. Therefore, the photoconversion effect for Rh6G might correlate to similarities in the chemical structure of these two dyes [42].

Due to the laser exposure, the intensity of capsules with PyB was reduced in the red channel but remained at the same level in the green channel (Fig. 3k,l). Therefore, the signal in the green channel was insufficient to detect for labeling purposes. The low stability of PyB dye was demonstrated previously [43], which correlates with the obtained results.

Additionally, λ-scans of capsules under 488 nm excitation before and after photoconversion were recorded (Fig. 3g–j,m). For all three dyes, a blue shift of the spectra was observed after laser irradiation. A similar phenomenon of absorbance spectra blue shift upon irradiation was demonstrated previously for RhB and Rh6G dyes [30,44]. To justify the observed effect for emission spectra, solutions of RhB and Rh6G dyes were prepared after thermal treatment with DS. The solutions were exposed to UV radiation to mimic the influence of the laser. The changes in the fluorescence spectra are shown in Fig. S5. The fluorescence spectrum of RhB demonstrated the growth of a left shoulder peak due to UV radiation. The fluorescence spectrum of Rh6G exhibited a blue shift upon UV irradiation. Therefore, the observed changes in the dyes fluorescence spectra after UV exposure support the blue shift of capsules λ-scans upon laser irradiation.

A blue shift of λ-scan in the case of PyB was also observed (Fig. 3m). Supposedly, a blue shift is present due to the decrease of the signal in the red channel. In other words, because there was a noticeable signal in the green channel before photoconversion, thereafter bleaching of the dye in the red channel, the green signal became more prominent. This effect resulted in a blue shift of capsule spectra. Nevertheless, since PyB did not demonstrate photoconversion ability, it was not studied further. Therefore, subsequent mass spectrometric studies were performed only for RhB and Rh6G.

### 3.6 Mass spectrometric study of RhB and Rh6G photoconversion mechanism

**Fig. 4:**
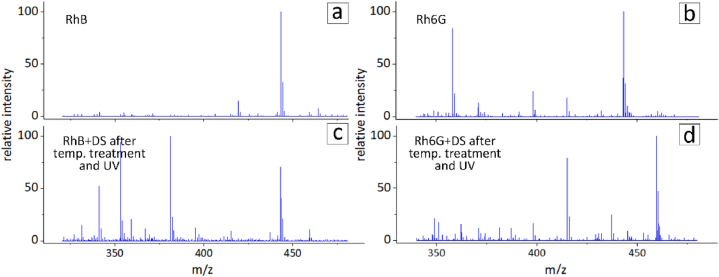
Mass spectra of RhB and Rh6G solutions (a, b) and RhB/Rh6G solutions with DS after thermal treatment and UV irradiation (c, d).

Previously, it was suggested that capsule photoconversion is possible due to RhB going through several deethylation steps under laser irradiation in combination with CNDs acting as a catalyst [20]. To verify this hypothesis, a mass spectrometry study of RhB and Rh6G aqueous solutions was performed. Dye solutions were prepared according to the same thermal treatment scheme as it was used for the capsules. Aqueous solutions of the dyes without thermal treatment were used as the controls. The mass spectrometry results obtained for the four prepared samples are shown in Fig. 4a–d.

According to the mass spectra, after UV irradiation other forms of RhB and Rh6G appeared with the removed ethylene groups. Values *m*/*z* and corresponding lost groups are summarized in Table 1. The numbers in brackets indicate the numbers of the ethylene groups. A similar deethylation effect was observed for the photooxidation of RhB intermediates [44] and the Rh6G dye [45]. The removal of ethylene groups leads to a change in the chemical structure of the dye. Such intermediates begin to emit in a shorter wavelength range, providing the basis for the desired photoconversion effect in polymer capsules. In addition to other studies [44,45], this blue spectral shift was also confirmed by the change in fluorescence spectra of the dyes after UV irradiation (Fig. S5). RhB exhibited noticeable growth of shoulder peaks at 536 nm, and Rh6G showed a 2 nm blue shift after UV irradiation. Thus, mass spectrometry confirmed that the spectral shifts of RhB and Rh6G in the capsules were due to the deethylation reaction.

**Table 1:** Interpretation of the removed groups of fragment ions of Rhodamine B and Rhodamine 6G after thermal treatment and UV irradiation.

Rhodamine B		Rhodamine 6G	
*m*/*z*	loss	*m*/*z*	loss
443.2		443.2	
415.2	C_2_H_4_	415.2	C_2_H_4_
387.2	C_2_H_4_ (2)	401.2	C_2_H_4_, CH_3_
359.2	C_2_H_4_ (3)	387.2	C_2_H_4_ (2)
331.3	C_2_H_4_ (4)	386.1	C_2_H_4_, CH_3_, NH_2_
		372.2	C_2_H_4_ (2), NH_2_
		359.2	C_2_H_4_, CH_3_, C_2_H_5_NH

### 3.7 Metabolic activity of the cells and uptake efficiency

At the next stage of the research, the influence of photoconvertible polymer capsules on two macrophage cell lines was investigated. It is well known that macrophages are hard to transfect. These cells recognize foreign nucleic acids and initiate an immune response to these molecules [18]. Polymer microcapsules do not lead to such effects, which makes them especially interesting for labeling macrophages as an alternative to transfection. It is essential to adjust the concentration of capsules added per cell to get the desired level of internalization and ensure sufficient cell viability.

**Fig. 5:**
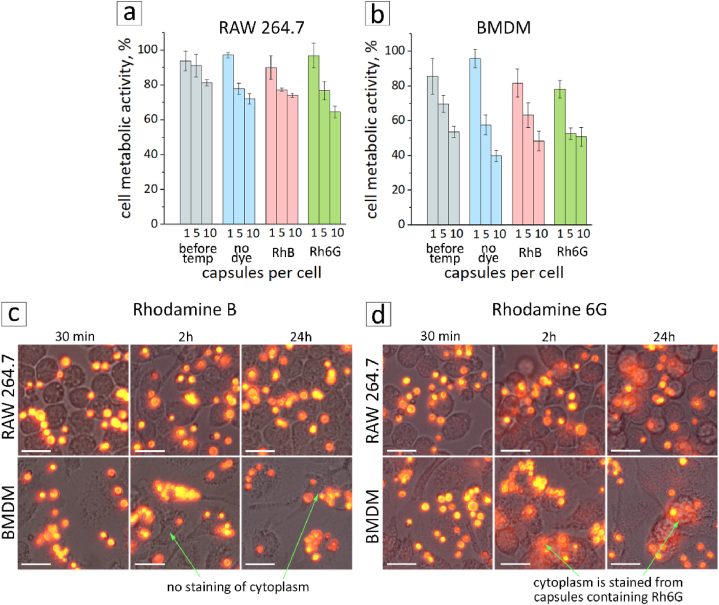
Assessment of the metabolic activity of RAW 264.7 (a) and BMDM (b) cells by adding empty, heat-treated empty, and heat-treated capsules with RhB or Rh6G dyes at 1, 5, and 10 capsules per cell. (c,d) Fluorescence microscopy images combined with brightfield images of RAW 264.7 and BMDM cells with 5 capsules/cell (RhB and Rh6G). Images were taken after the addition of capsules, 0.5, 2, and 24 h later (scale bar is 20 μm).

MTT assay was performed to investigate cell metabolic activity by adding 1, 5, and 10 capsules per cell (Fig. 5a and b). Four samples were used: control capsules before thermal treatment, and capsules after thermal treatment with RhB, Rh6G, and without the dyes. According to the obtained data, the cell viability of RAW 264.7 remained around 90 % when five control capsules per cell were added. All thermally treated capsules added at the same concentration resulted in approximately 80 % cell viability. This indicates that the capsules after thermal treatment are still safe to use in cells. In addition, no significant difference was observed for the capsules after thermal treatment with and without the dyes. This indicated that the dye did not cause additional cytotoxicity. BMDM cells appeared to be more sensitive to capsules of all four types. Their metabolic activity was reduced by approximately 40 % when capsules were added at a concentration of 5 capsules/cell. Supposedly, BMDM cells are more sensitive to capsules because these cells are a primary culture with a low division rate [46,47]. At the same time, RAW 264.7 is an immortalized cell line with an average doubling period of approximately 12 h and faster metabolism [48]. Owing to these differences, the two cell lines exhibit different sensitivities to drugs. Other studies also have demonstrated that BMDM cells are more susceptible to the toxic effects of foreign substances [5].

While the capsules containing encapsulated Rh6G were added to the cells for 24 h, both cell types were noted to be stained with Rh6G indicating possible leakage from the capsules. One can see in Fig. 5d that the cytoplasm began to emit in the red channel after 2 h of incubation. At the same time, the capsules containing RhB did not exhibit this effect (Fig. 5c) while internalized by the cells.

Aqueous solutions of RhB and Rh6G were prepared for a clearer understanding and explanation of this phenomenon. Dye solutions were mixed with either water or BSA aqueous solution and placed on a thermoshaker at 37 °C for 2 h to mimic the interaction of dyes with the internal environment of the cells. The fluorescence intensity of Rh6G increased significantly when the BSA solution was added (Fig. S6). Nevertheless, the intensity of RhB remained almost at the same level. This corresponds with the data, obtained earlier for rhodamine dyes [49]. This effect can be explained by the fact that the binding constant of BSA for Rh6G is higher than that for RhB. As a result, the macrophage cytoplasm could have a pronounced fluorescent signal from Rh6G, not due to faster dye release (compared to RhB), but due to a higher binding constant with increasing quantum yield. Therefore, the uptake efficiency was determined further only for capsules with RhB, even though promising MTT results were obtained for the Rh6G dye as well.

**Fig. 6:**
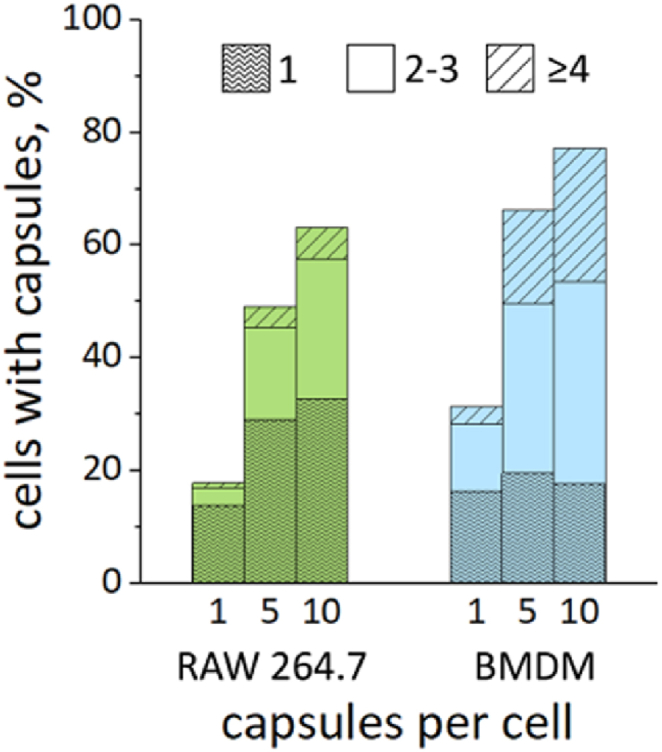
Dependence of the number of cells with internalized capsules containing RhB on the number of added capsules.

In the uptake experiment, the capsules at the ratio of 1, 5, and 10 per cell were added to the cells (Fig. 6). For RAW 264.7, 50 % of the cells internalized capsules when 5 capsules/cell were added. BMDM uptake efficiency was 65 % under the same conditions. In addition, in the MTT assay, their metabolic activity decreased to approximately 60 %, which was lower than that of RAW 264.7. This may be due to the more pronounced phagocytic activity of the primary culture of BMBM cells than that of the permanent RAW 264.7 culture [50]. Also, macrophages are an important part of the immune system because these cells are the first line of defense against bacteria and foreign particles [51]. Their metabolic profiles can change in response to the microenvironment, shaping their activation state and function [52]. Therefore, the decrease in metabolic activity of BMDM macrophages might correlate with the high uptake instead of the cytotoxicity of the capsules. Overall, 5 capsules/cell was chosen as the optimal concentration for further research.

### 3.8 Effect of microcapsules on cells mobility and photoconversion of capsules inside the cells

**Fig. 7:**
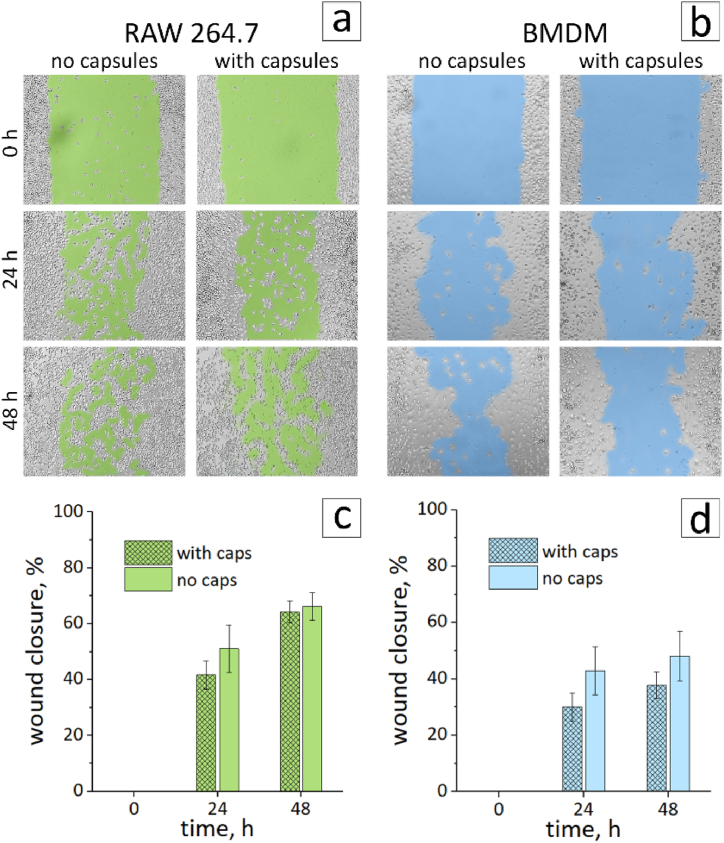
Bright-field microscopy images of the wound closure for RAW 264.7 (a) and BMDM (b) with and without the capsules. Graph of wound closure over time for RAW 264.7 (c) and BMDM (d).

In order to examine the effect of capsules on cell mobility, a wound-healing assay was performed. Capsules were added to the cells at a concentration of 5 capsules/cell, followed by scratch assay. Changes in the wound area were observed for 48 h (Fig. 7a–d). The graph reflects the percentage of wound closure over time, where 100 % represents the complete disappearance of the wound. Capsules affected the mobility of RAW 264.7 only at first 24 h and this effect levelled off at 48 h. BMDM cells were more sensitive and the addition of capsules led to a slight decrease in mobility at both 24 and 48 h. In general, capsules have an insignificant effect on cell mobility. It corresponds with the data of other authors describing the effect of polyarginine/DS capsules on macrophage mobility [5].

**Fig. 8:**
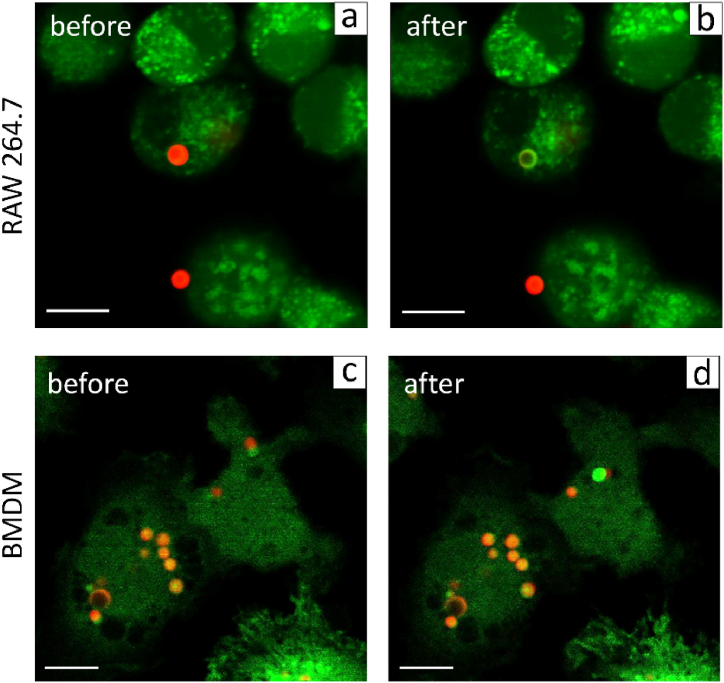
CLSM images of RAW (a,b) 264.7 and BMDM (c,d) cells before and after photoconversion of capsules inside the cells (scale bar is 10 μm).

To demonstrate the possibility of macrophage labeling and tracking, capsules containing RhB were added to the cells (5 capsules/cell). The capsules showed bright fluorescence after internalization, and the cells retained their characteristic morphology (Fig. 8). According to the procedure described earlier, capsules with RhB were converted using a 561 nm laser of the confocal microscope. This led to the immediate appearance of a fluorescence signal from this capsule in the range 505–540 nm (488 nm laser excitation), which became green in the confocal image. Neither cell line changed its morphology. All marked cells remained highly viable after the procedure; therefore, laser application did not negatively affect them. This result correlates with the data obtained earlier for other cell types, such as HeLa and C2C12 cells [20]. Therefore, polymer photoconvertible capsules with RhB could be successfully used as tracking labels for macrophages.

## 4 Conclusions

A comprehensive protocol for the synthesis of fluorescent polymer capsules in the PVA gel was elaborated. This protocol allowed to increase the yield of capsules from one synthesis by at least 250 times. This method also offers ease of replication and enables the encapsulation of various dyes within capsules. The encapsulated dyes exhibited robust fluorescence after thermal treatment. Also, the fluorescence intensity of the capsules containing the dyes varied depending on their fluorescence spectra and confocal detection regions. Nevertheless, during the bleaching assay, fluorescein, acridine yellow, acridine orange, and thiazine red dyes displayed low photostability. The rest of the encapsulated dyes: rhodamine B, rhodamine 6G, and pyronin B demonstrated high photostability. During further studies, rhodamine B and rhodamine 6G were found to have remarkable photoconversion properties, while pyronin B did not possess this characteristic. This photoconversion effect was clearly evident when comparing the fluorescence intensities before and after the procedure. A significant decrease was observed in the 580−620/670 nm detection range and a noticeable increase was seen in the 505−540 nm detection range. Mass spectrometry analysis revealed that the rhodamine dyes underwent several deethylation steps under conditions similar to those of the photoconversion procedure. Consequently, new compounds with blue spectral shifts were formed, enabling the visualization of the capsules in the green channel following laser irradiation [44,45]. This supports the proposed hypothesis regarding the presence of a deethylation reaction during the photoconversion process.

Both rhodamine B and rhodamine 6G-containing capsules exhibited similar effects on the metabolic activity of the investigated cell lines. Nevertheless, rhodamine 6G caused undesirable staining of macrophage cytoplasm. It might be due to the enhanced fluorescence that occurs upon dye-protein binding [49]. Therefore, rhodamine B became a more suitable choice for further experiments on macrophages. Despite this, capsules containing rhodamine 6G still may be useful for tracking cells with lower metabolic activity, such as mesenchymal stem cells [53], neuron cells [54], fibroblasts [55], and others. RAW 264.7 and bone marrow-derived macrophages were chosen to study the efficacy and safety of our polymer labels. A high uptake of capsules with minimal impact on cell viability was demonstrated at the added capsule-to-cell ratio of 5:1. Moreover, capsule internalization had a negligible effect on cell mobility and did not alter cell morphology. Successful photoconversion of capsules containing rhodamine B was achieved within the cells. It highlighted the potential of these photoconvertible capsules as an attractive alternative to green fluorescent proteins for macrophage labeling. Since macrophages are also known to not exchange internalized capsules [5], such labels become even more promising for their tracking.

In future studies, photoconvertible polymer capsules could open new possibilities for unique coding systems through different combinations of labels within individual cells. Remarkably, these capsules can persist within the cells for several days while retaining their bright and stable fluorescence [53]. The absence of genetic modification when using the developed labels for marking cells eliminates the risk of spontaneous mutations. Therefore, our labels can be considered for personalized medicine, for example, cancer treatment and stem cell therapy.

## Ethics statement

The animal study protocol was approved by the Ethics Committee of the Koltzov Institute of Developmental Biology of the Russian Academy of Sciences (protocol № 55; December 9, 2021).

## Funding

This work was supported by the 10.13039/501100006769Russian Science Foundation (grant number 22-15-00292).

## Data availability statement

Data will be made available on request.

## CRediT authorship contribution statement

**Zhanna V. Kozyreva:** Writing – original draft, Visualization, Investigation, Formal analysis, Data curation. **Polina A. Demina:** Visualization, Methodology, Investigation, Conceptualization. **Anastasiia Yu Sapach:** Methodology, Investigation. **Daria A. Terentyeva:** Investigation. **Olga I. Gusliakova:** Methodology, Investigation, Formal analysis. **Anna M. Abramova:** Investigation. **Irina Yu Goryacheva:** Writing – original draft, Validation, Methodology, Conceptualization. **Daria B. Trushina:** Formal analysis. **Gleb B. Sukhorukov:** Writing – review & editing, Supervision, Funding acquisition, Conceptualization. **Olga A. Sindeeva:** Writing – review & editing, Supervision, Project administration, Conceptualization.

## Declaration of generative AI and AI-assisted technologies in the writing process

During the preparation of this work, the authors used Grammarly in order to improve language and readability. After using this tool, the authors reviewed and edited the content as needed and take full responsibility for the content of the publication.

## Declaration of competing interest

The authors declare that they have no known competing financial interests or personal relationships that could have appeared to influence the work reported in this paper.
