# Supplementary material for: Multiple dyes applications for fluorescent convertible polymer capsules as macrophages tracking labels
Source: Heliyon. 2024 May 5;10(10):e30680. doi: 10.1016/j.heliyon.2024.e30680 (PMC11133507; doi:10.1016/j.heliyon.2024.e30680)
Supplement: Multimedia component 1 [file mmc1.pdf]

# Supporting information

## Multiple Dyes Applications for Fluorescent Convertible Polymer Capsules as Macrophages Tracking Labels

Zhanna V. Kozyreva[1], Polina A. Demina[2], Anastasiia Yu. Sapach[1], Daria A. Terentyeva[1], Olga I. Gusliakova[1,2], Anna M. Abramova[2], Irina Yu. Goryacheva[1,2], Daria B. Trushina[3], Gleb B. Sukhorukov[1,4,*], and Olga A. Sindeeva[1]

[1] Skolkovo Institute of Science and Technology, Moscow, 121205, Russia

[2] Saratov State University, Saratov, 410012, Russia

[3] Sechenov University, Moscow, 119991, Russia

[4] Queen Mary University of London, London, E1 4NS, UK

*Corresponding author: g.sukhorukov@skoltech.ru (G.B.S.)

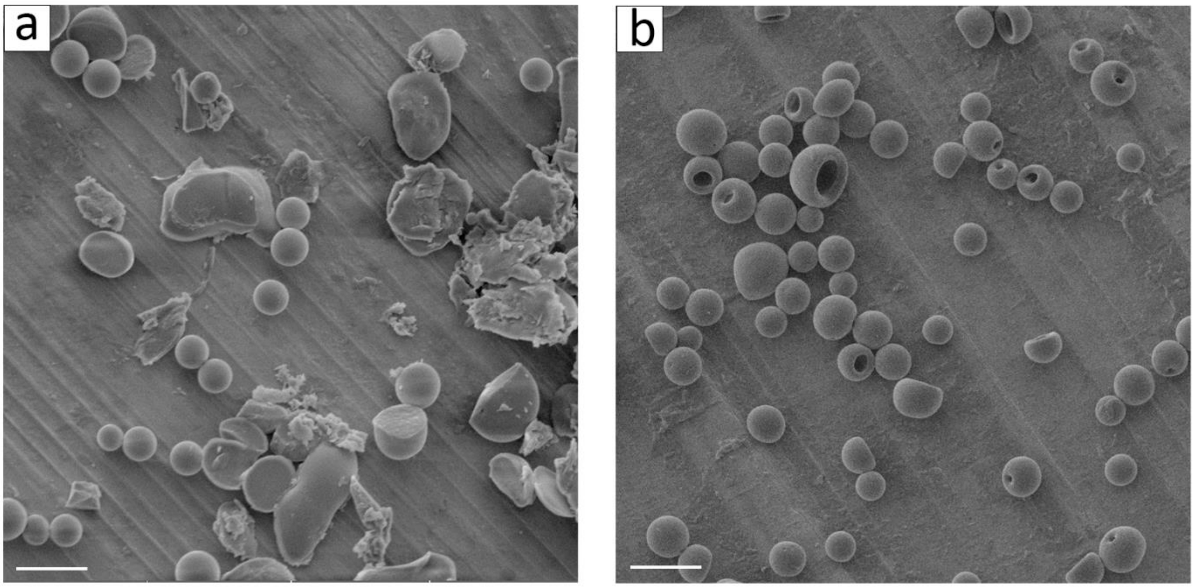


Figure S1. SEM images of polymeric microcapsules after thermal treatment in water (a) and PVA gel (b) (scale bar is 5 μm).

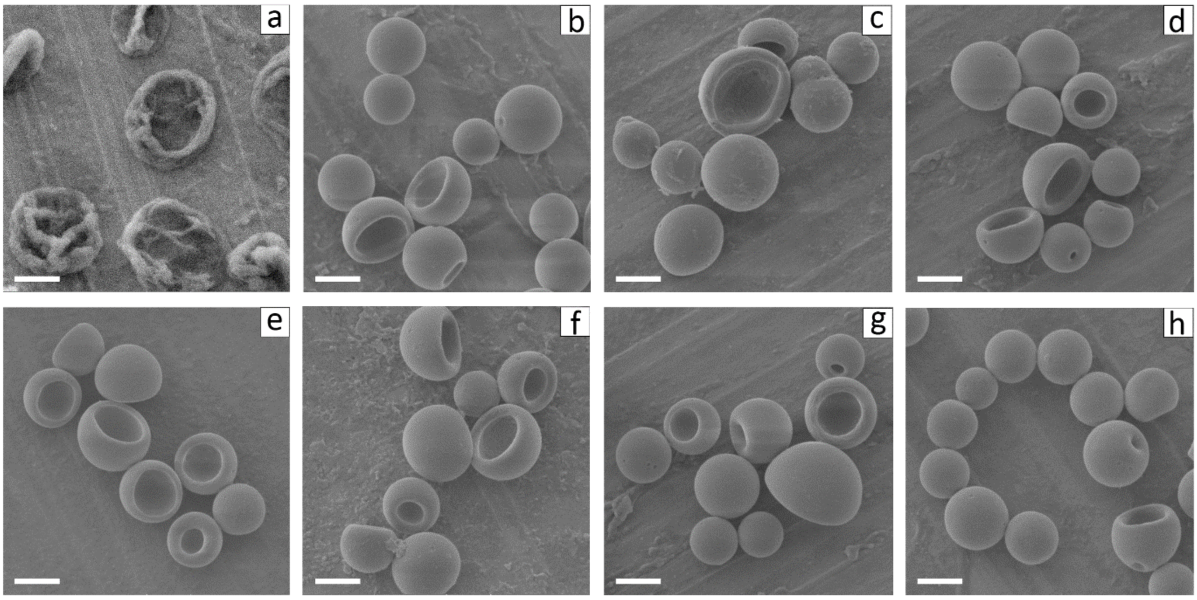


Figure S2. SEM images of polymeric microcapsules before (a) and after thermal treatment in PVA gel with Rhodamine B (b), Rhodamine 6G (c), Pyronin B (d), Fluorescein (e), Acridine Yellow (f), Acridine Orange (g), and Thiazine Red (h) (scale bar is 2 μm).

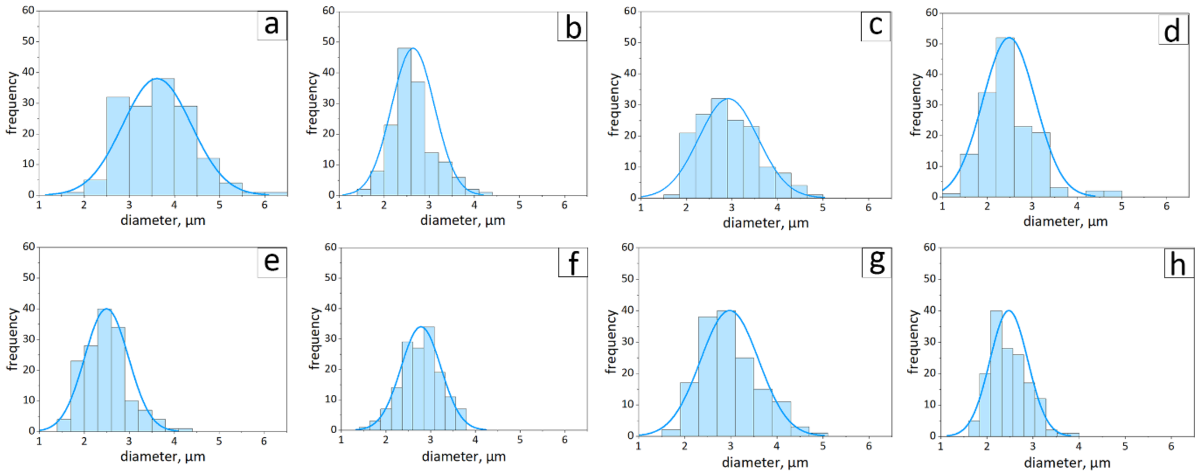


Figure S3. Histograms of polymeric microcapsules size distribution before (a) and after thermal treatment in PVA gel. Microcapsules are loaded with Rhodamine B (b), Rhodamine 6G (c), Pyronin B (d), Fluorescein (e), Acridine Yellow (f), Acridine Orange (g), and Thiazine Red (h).

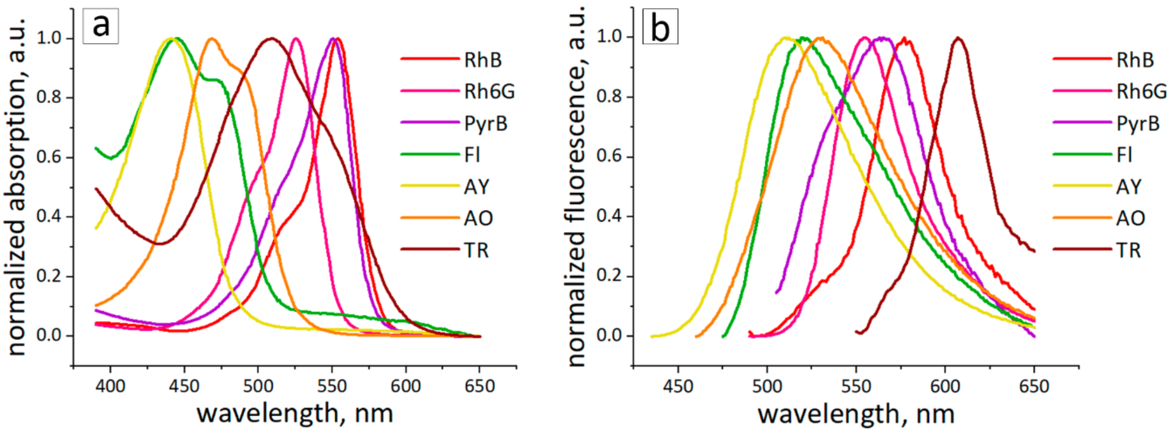


Figure S4. Normalized absorption (a) and fluorescence (b) spectra of the dye solutions. For fluorescence detection of RhB and Rh6G: λex = 455 nm, detection range 490-650 nm, for PyB λex = 470 nm, detection range 505-650 nm, for Fl λex = 440 nm, detection range 475-650 nm, for AY λex = 400 nm, detection range 435-650 nm, for AO λex = 425 nm, detection range 460-650 nm, for TR λex = 405 nm, detection range 540-650 nm.

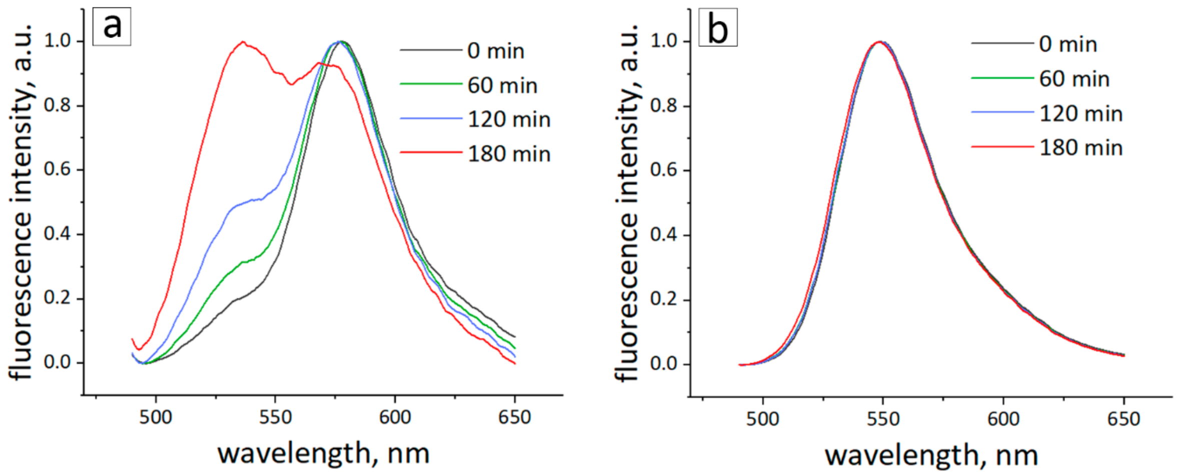


Figure S5. Normalized fluorescence spectra of the RhB (a) and Rh6G (b) dye solutions after thermal treatment with DS before and after UV irradiation for 60, 120, and 180 minutes.

The changes in the fluorescence spectra of RhB and Rh6G solutions with CNDs, obtained from DS after thermal treatment are presented in Figure S5, depending on the duration of UV irradiation. The RhB spectrum showed a noticeable growth of the shoulder peak. The Rh6G spectrum experienced a 2 nm blue shift.

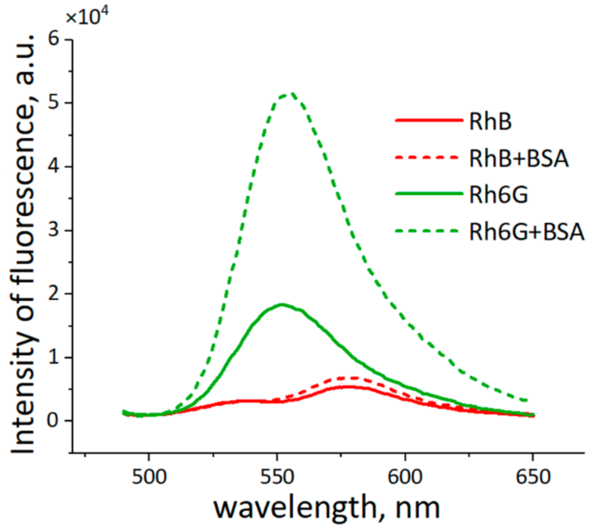


Figure S6. Fluorescence spectra of RhB and Rh6G solutions with and without BSA after 2 hours in the thermoshaker at 37 °C.
